# Supplementary material for: Colonic spatial single-cell proteomics and murine models link mitochondrial dysfunction to dimeric IgA-secreting plasma cell deficiency in Crohn’s disease
Source: Nat Commun. 2026 Feb 12;17:1590. doi: 10.1038/s41467-026-69069-w (PMC12901160; doi:10.1038/s41467-026-69069-w)
Supplement: Supplementary file 1 — Supplementary Information [file 41467_2026_69069_MOESM1_ESM.pdf]

Colonic spatial single-cell proteomics and murine models link mitochondrial dysfunction to dimeric IgA-secreting plasma cell deficiency in Crohn's disease

Annika Raschdorf, Larissa Nogueira de Almeida, Philipp Solbach, Martha M Kirstein, Jens U Marquardt, Franziska Schmelter, Ulrich L Günther, Heidi Schlichting, Maren Hicken, Lea Christiansen, Miriam Wiestler, Hauke C Tews, Dominik Bettenworth, OUTLIVE-CRC consortium, Matthias Peipp, Thomas Valerius, Mohab Ragab, Thorben Sauer, Timo Gemoll, Marc Ehlers, Philip Rosenstiel, Rudolf Armin Manz, Axel Künstner, Hauke Busch, Christian Sina, Stefanie Derer

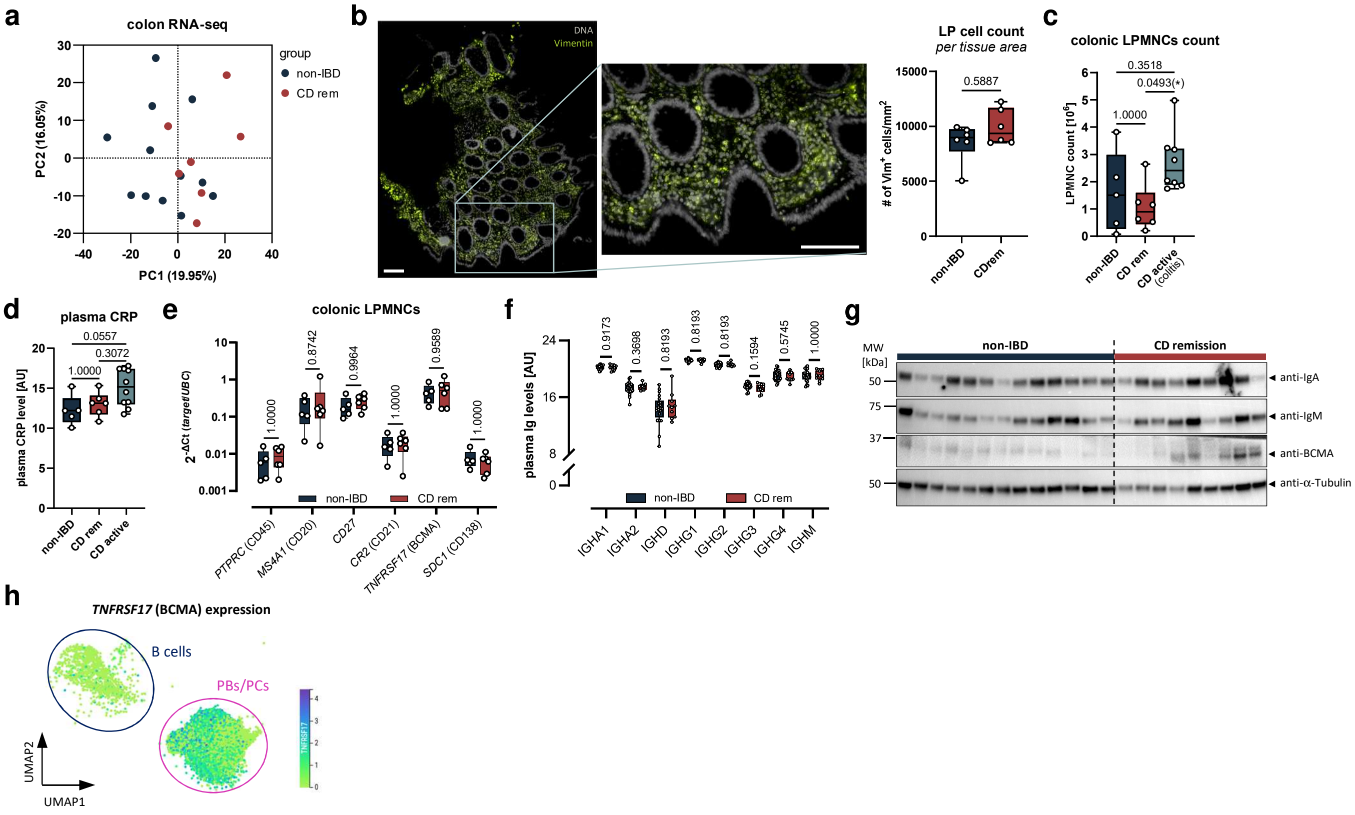

Supplementary Fig. 1 | Characterization of colonic LPMNCs and Ig levels in CD^{rem} patients and non-IBD controls. (a) PCA displaying the colonic transcriptomic profile (standardized TPM values of the 1,000 most variable genes) of CD^{rem} patients (n=7) and non-IBD controls (n=12). **(b)** Representative image of Vimentin staining (yellowish green) of the LP cells in a colonic biopsy section in the CosMx^{TM} SMI and Vim^{+} cell count per tissue area [mm^{2}] for CD^{rem} patients (n=6) and non-IBD controls (n=6). Scale bars: 100 µm. **(c)** Cell count of isolated colonic LPMNCs (CD^{rem} n=6, CD^{active} n=8, non-IBD n=5). **(d)** Plasma CRP levels were determined using LC-MS proteomics (CD^{rem} n=6, CD^{active} n=8, non-IBD n=5). **(e)** Expression of not significantly regulated B-cell markers in the isolated colonic LPMNCs of CD^{rem} (n=6) and non-IBD (n=5) was quantified using RT-qPCR. **(f)** Plasma Ig levels of CD^{rem} patients (n=10) and non-IBD controls (n=21) were quantified using LC-MS proteomics. **(g)** Detection of colonic IgA, IgM, and BCMA in CD^{rem} (n=9) and non-IBD (n=13) *via* Western blotting. **(h)** Single-cell transcriptomics data from the Human Gut Cell Atlas[1] display specific expression of *TNFRSF17* (BCMA) in human colonic PBs and PCs, but not B cells. **(b)** and **(f)** Two-tailed (Multiple) Mann-Whitney U test, **(c)** and **(d)** Kruskal-Wallis test with Dunn's multiple comparisons test, **(e)** Two-way ANOVA with Šídák's multiple comparisons test. Box plots depict the median and IQR with whiskers indicating the range. AU, arbitrary units; IQR, interquartile range.

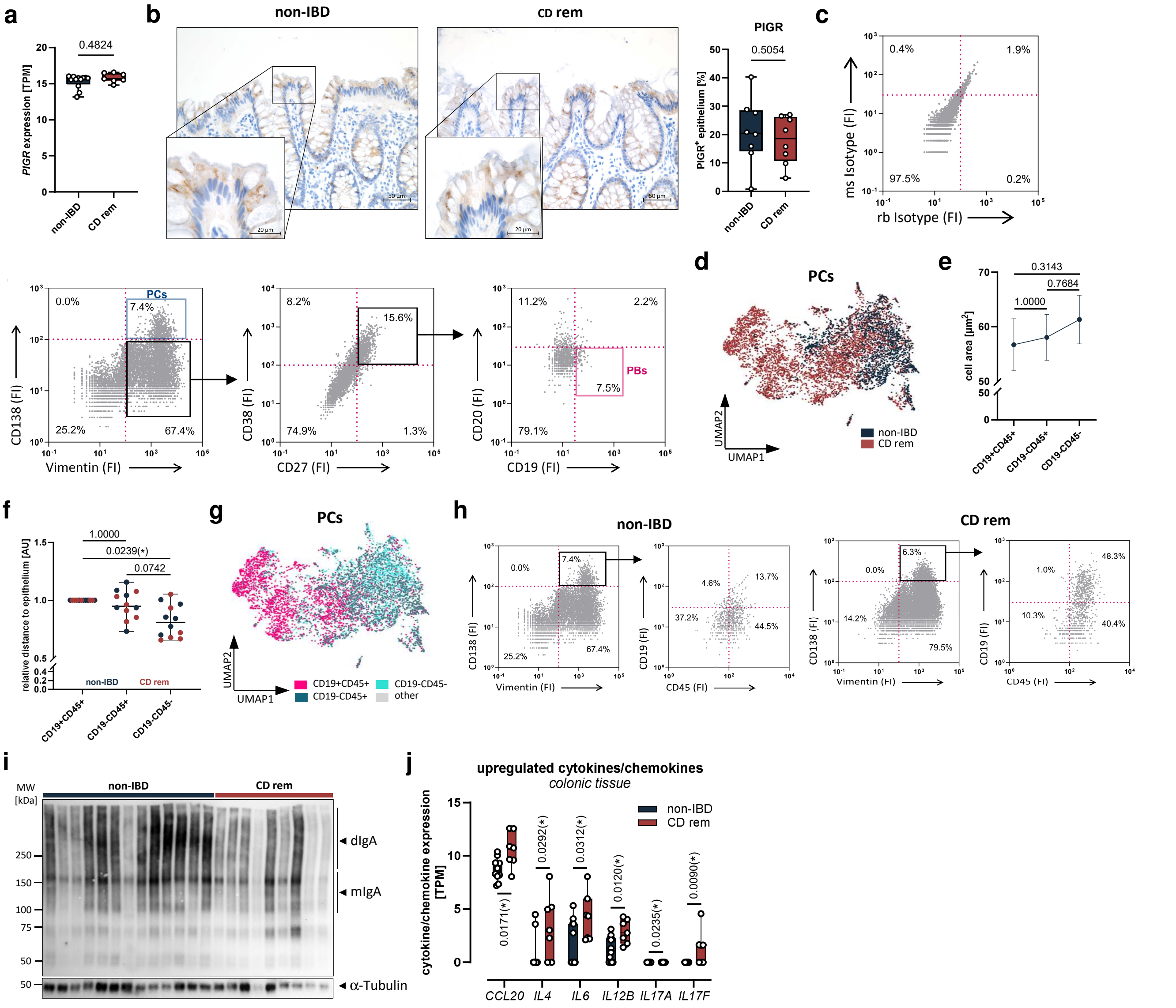

Supplementary Fig. 2 | Analysis of colonic PIGR levels, PCs and dIgA in CD^{rem} patients. (a) Colonic expression of *PIGR* in CD^{rem} (n=7) and non-IBD (n=12) from RNA-seq data is displayed as transcripts per million (TPM). **(b)** IHC staining of colonic pIgR (CD^{rem} n=8, non-IBD n=8) (left) and quantification of epithelial pIgR (right). **(c)** Separation strategy of PBs and PCs in spatial single-cell proteomics data (bottom) using positivity cut-offs of 30 and 100 for mouse- or rabbit-derived antibodies, respectively (top). **(d)** UMAP of total colonic $CD138^+$ PCs of the second SSCP experiment (CD^{rem} n=3, non-IBD n=3). **(e)** Median cell area of mucosal PC subpopulations of six non-IBD controls was determined using cell segmentation of SSCP. **(f)** Median distance of mucosal PC populations to the colonic epithelium in CD^{rem} patients (n=6) and non-IBD controls (n=6) was determined using SSCP and displayed as relative values compared to $CD19^+CD45^+$ PCs per patient. **(g)** UMAP displaying the colonic $CD138^+$ PC subtypes of CD^{rem} patients (n=3) and non-IBD controls (n=3) of the second SSCP experiment. **(h)** Representative scatter plots of PCs (Vim^+CD138^+) from total colonic mucosal cells of a non-IBD patient (left) and a CD^{rem} patient (right) analyzed for the expression of CD19 and CD45. **(i)** Detection of mIgA and dIgA in colonic biopsies of CD^{rem} patients (n=9) and non-IBD controls (n=13) via Western blotting. **(j)** Upregulated expression of six cytokines/chemokines was identified in the colonic mucosa of CD^{rem} patients (n=7) versus non-IBD controls (n=12) using RNA-seq. **(a)**, **(b)**, and **(j)** Two-tailed (Multiple) Mann-Whitney U test, **(e)** Kruskal-Wallis test with Dunn's multiple comparisons test, **(f)** Friedman test with Dunn's multiple comparisons test. Box plots depict the median and IQR with whiskers indicating the range. *P≤.05. AU, arbitrary units; FI, fluorescence intensity; IQR, interquartile range.

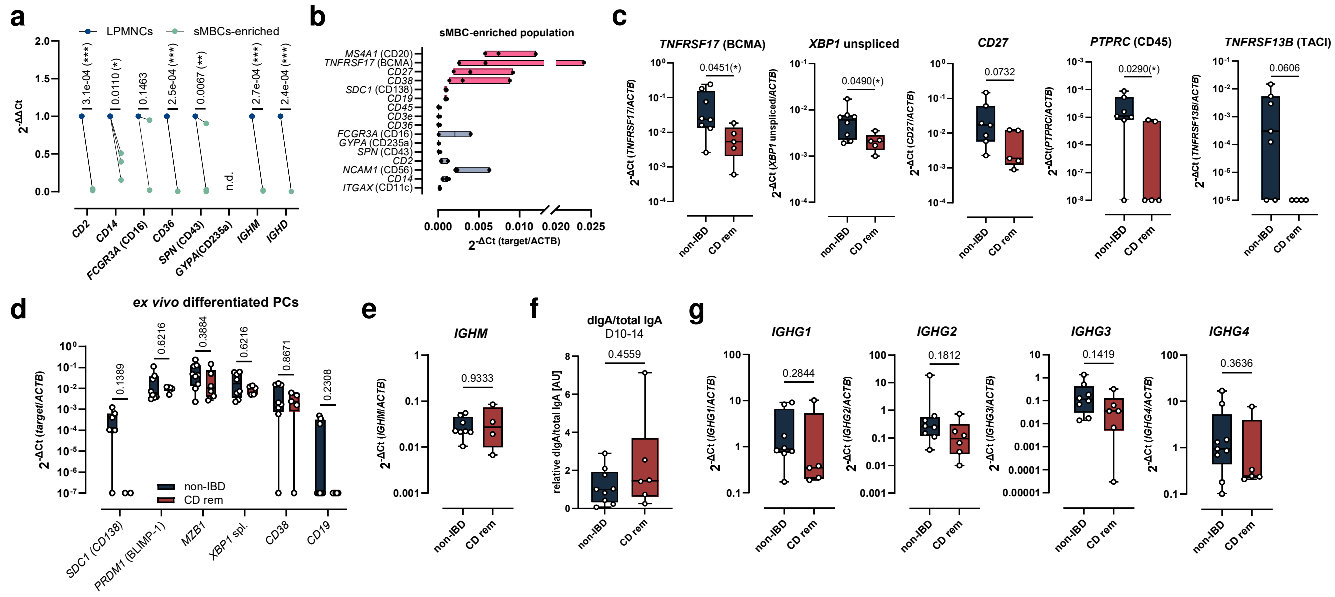

Supplementary Fig. 3 | Phenotyping of ex vivo differentiated colonic PCs. (a) Depletion of cells positive for CD2, CD14, CD16, CD36, CD43, CD235a, IgM, or IgD via MACS was verified by RT-qPCR using LPMNCs and isolated sMBCs (n=3 each). Expression of each target gene was normalized to the housekeeper *UBC* ($2^{-\Delta Ct}$) and depicted as relative mRNA expression to the respective LPMNCs ($2^{-\Delta\Delta Ct}$). **(b)** Expression of B-cell related transcripts (pink) and transcripts present in T cells, natural killer (NK) cells, monocytes, macrophages, erythrocytes, dendritic cells (DCs) or granulocytes (bluish gray) in the sMBC-enriched cell population (n=2-3) was determined using RT-qPCR. **(c)** Expression of altered (P<0.1) B cell/PC markers in ex vivo differentiated colonic PCs from CD^{rem} patients (*TNFRSF17*/*XBP1* unspliced/*CD27*/*PTPRC* n=5, *TNFRSF13B* n=4) and non-IBD controls (*TNFRSF17*/*XBP1* unspliced n=8, *CD27*/*PTPRC*/*TNFRSF13B* n=7). **(d)** Expression of not significantly regulated PC markers in CD^{rem}- (*SDC1* n=2, *PRDM1*/*XBP1* spl./*CD38*/*CD19* n=5, *MZB1* n=6) and non-IBD-derived (*SDC1* n=7, *PRDM1*/*XBP1* spl./*CD38*/*CD19* n=8, *MZB1* n=9) ex vivo differentiated colonic PCs on Day 14 was quantified using RT-qPCR. **(e)** Expression of *IGHM* in ex vivo differentiated CD^{rem}- (n=4) or non-IBD-derived (n=8) colonic PCs. **(f)** Ratio of dIgA/total IgA in the supernatant of CD^{rem}- (n=6) versus non-IBD-derived (n=9) ex vivo differentiated colonic PCs between Day10-14. **(g)** Expression of the IgG subclass genes in ex vivo differentiated CD^{rem}- (*IGHG1*/*4* n=5, *IGHG2*/*3* n=6) and non-IBD-derived (*IGHG1*/*2*/*3* n=8, *IGHG4* n=9) colonic PCs. **(a)** Two-way ANOVA with Bonferroni's multiple comparisons test, **(c)**-**(g)** Two-tailed (Multiple) Mann-Whitney U test. Box plots depict the median and IQR with whiskers indicating the range. *P≤.05, **P≤.01, ***P≤.001. AU, arbitrary units; IQR, interquartile range; n.d., not detected.

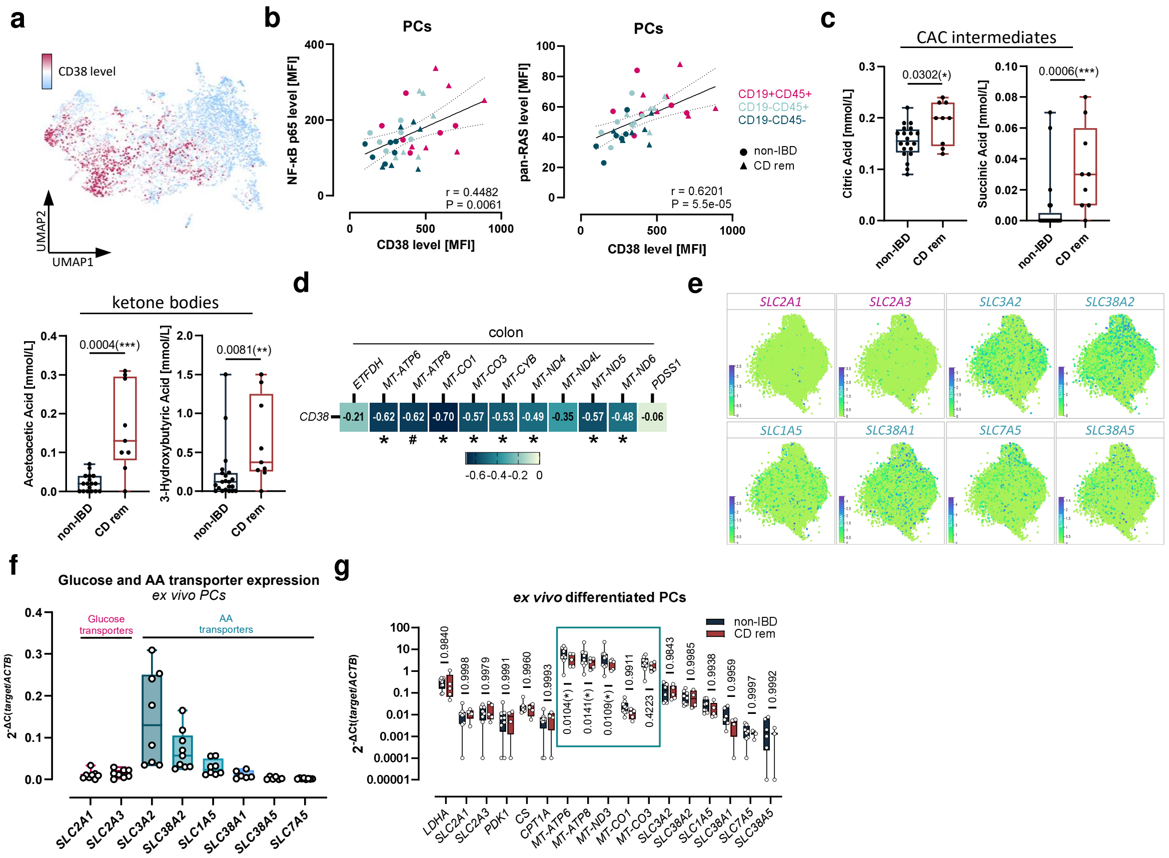

Supplementary Fig. 4 | Analysis of CD38 expression and mitochondrial metabolism in CD^{rem}. (a) UMAP displaying the level of CD38 within all colonic PCs of CD^{rem} patients (n=3) and non-IBD controls (n=3) of the second SSCP experiment. **(b)** Correlation of CD38 levels with NF-κB p65 (left) and pan-RAS levels (right) across all three colonic CD138^{+} PC subpopulations in CD^{rem} (triangles, n=6) and non-IBD (circles, n=6). **(c)** Plasma levels of citric acid, succinic acid, acetoacetic acid, and 3-hydroxybutyric acid in CD^{rem} patients (n=9) compared to non-IBD controls (n=21). **(d)** Correlation of colonic CD38 expression with significantly decreased mitochondria-related transcripts (Fig. 6f) of CD^{rem} patients (n=7) and non-IBD controls (n=12) displaying Spearman correlation coefficients. **(e)** Glucose transporter (magenta) and amino acid transporter (subunit) (blue) expression in single-cell transcriptomics data of colonic IgA^{+} PCs from healthy adults, publicly available in the Human Gut Cell Atlas[1]. **(f)** Expression of various glucose and amino acid transporters was quantified in non-IBD-derived ex vivo differentiated colonic PCs (n=9) using RT-qPCR. **(g)** Expression levels of metabolic enzyme(s) (subunits) and nutrient transporters were determined in CD^{rem}- (*LDHA*/*SLC2A1*/*SLC2A3*/*MT-ATP6*/*MT-ATP8*/*MT-ND3*/*MT-CO1*/*MT-CO3*/*SLC3A2*/*SLC1A5* n=5, *PDK1*/*CS*/*CPT1A*/*SLC38A2*/*SLC38A1* n=4, *SLC7A5*/*SLC38A5* n=3) and non-IBD-derived (*LDHA*/*PDK1*/*CPT1A*/*MT-ATP6*/*MT-ATP8*/*MT-ND3*/*MT-CO1*/*MT-CO3*/*SLC38A2* n=9, *SLC2A1*/*SLC2A3*/ *SLC3A2*/*SLC1A5*/*SLC7A5* n=8, *CS* n=7, *SLC38A1*/*SLC38A5* n=6) ex vivo differentiated colonic PCs on Day 14 using RT-qPCR with mitochondrial genes highlighted in a green box. **(b)** Spearman correlation and linear regression with 95% confidence interval (dashed lines), **(c)** Two-tailed Mann-Whitney U test, **(d)** Spearman correlation, **(g)** Two-way ANOVA with Fisher's least significant difference (LSD) test. Box plots depict the median and IQR with whiskers indicating the range. **P*≤.05, ***P*≤.01, ****P*≤.001. IQR, interquartile range; MFI, median fluorescence intensity.

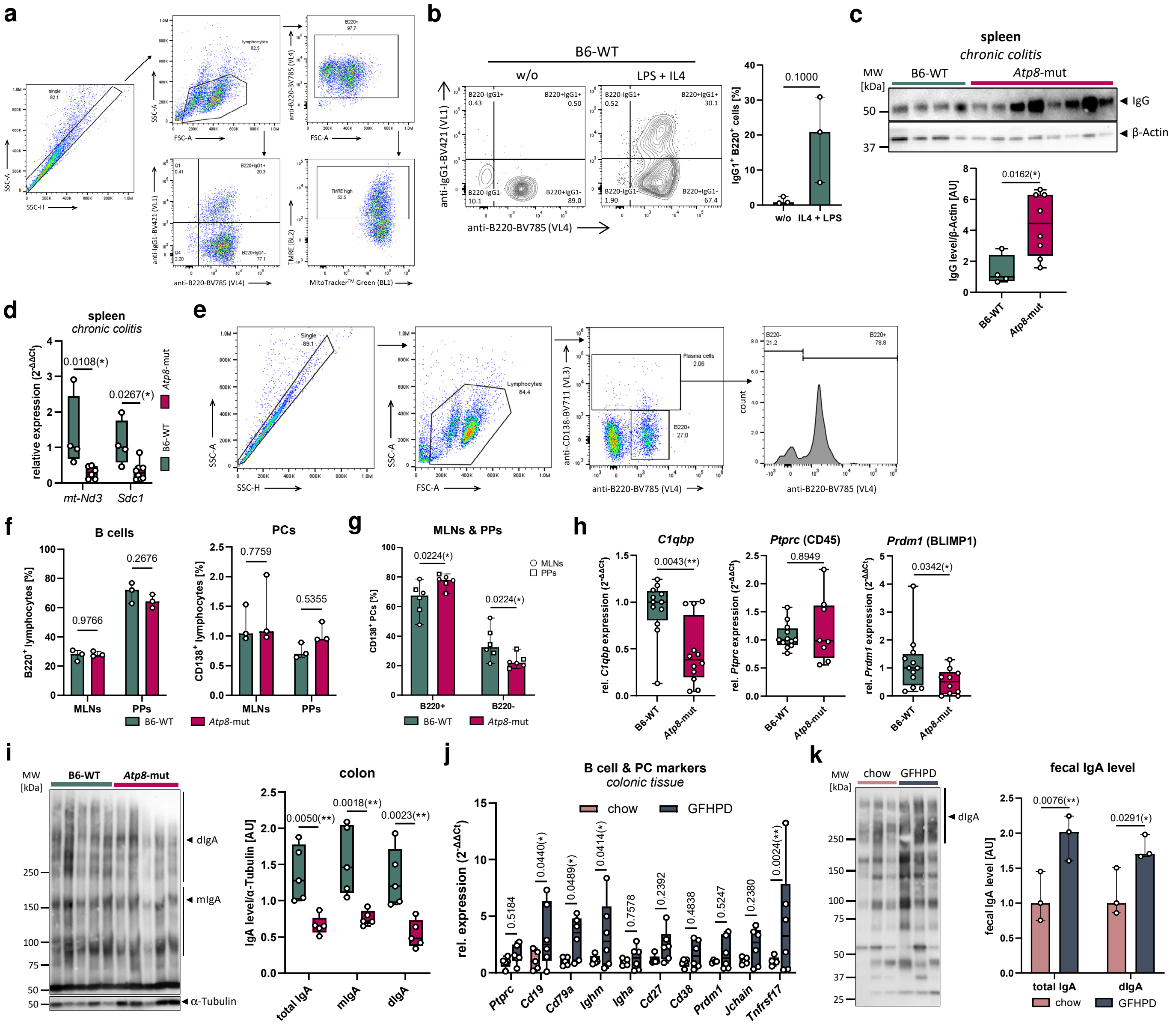

Supplementary Fig. 5 | Comparison of the intestinal B-cell/PC compartment between *Atp8*-mutant and B6-WT mice. (a) After exclusion of doublets (SSC-A versus SSC-H), single splenic lymphocytes after four days of cultivation were gated based on FSC-A versus SSC-A to remove debris and dead cells, followed by analysis of either B220$^+$IgG1$^+$ cells or TMRE^{high} cells within the B220$^+$ population. **(b)** Representative contour plots of B220 and IgG1 on splenic B cells of B6-WT mice (left) show the generation of IgG1$^+$ B cells upon stimulation with LPS+IL4 (n=3; right) from two independent experiments. **(c)** Quantification of splenic IgG levels in *Atp8*-mutant (n=8) and B6-WT mice (n=4) under chronic DSS-induced colitis from two independent sampling rounds using Western blot. **(d)** Relative expression levels of *mt-Nd3* and *Sdc1* in the spleen of *Atp8*-mutant (*mt-Nd3* n=6, *Sdc1* n=8) compared to B6-WT mice (n=4; median set to 1) under chronic DSS-induced colitis were determined via RT-qPCR across two independent sampling rounds. **(e)** After exclusion of doublets (SSC-A versus SSC-H), single lymphocytes from MLNs and PPs were gated based on FSC-A versus SSC-A to remove debris and dead cells. B cells and PCs were subsequently identified as B220$^+$ and CD138$^+$ cells, respectively. **(f)** Percentages of B220$^+$ B cells (left) and CD138$^+$ PCs (right) in the lymphocytes isolated from MLNs and PPs of *Atp8*-mutant (n=3) and B6-WT mice (n=3) in two separate experiments. **(g)** Percentages of B220$^+$ and B220$^-$ CD138$^+$ PCs isolated from the MLNs (circles) and PPs (squares) of *Atp8*-mutant (n=3) and B6-WT mice (n=3) in two independent experiments. **(h)** Expression of *C1qbp*, *Ptprc*, and *Prdm1* mRNA was quantified in colonic tissue of *Atp8*-mutant (*C1qbp* n=12, *Ptprc* n=8, *Prdm1* n=10) and B6-WT mice (n=12) via RT-qPCR. Data were normalized to *Actb* and are displayed as values relative to B6-WT mice for each of the three sampling rounds. **(i)** Quantification via Western blotting indicated reduced total IgA, mIgA, and dIgA in colonic samples from male *Atp8*-mutant (n=5) compared to B6-WT mice (n=5) from three different sampling rounds. **(j)** Colonic expression of different B-cell and PC markers was quantified for GFHPD-fed (n=6) and chow diet-fed mice (n=5) via RT-qPCR. Data are displayed as relative values to chow diet-fed mice for two independent experiments. **(k)** Quantification of fecal IgA levels in mice after 10 weeks of nutritional intervention (chow diet n=3, GFHPD n=3) using non-reducing Western blotting. **(b)**-**(d)**, and **(h)** Two-tailed (Multiple) Mann-Whitney U test, **(f)**, **(g)**, **(i)**, and **(k)** Two-way ANOVA with Šídák's multiple comparisons test (f and i) or Fisher's least significant difference (LSD) test (g and k), **(j)** Mixed-effects model with Fisher's LSD test. Box plots depict the median and IQR with whiskers indicating the range. Bar graphs display the median with range. **P*≤.05, ***P*≤.01. AU, arbitrary units; IQR, interquartile range.

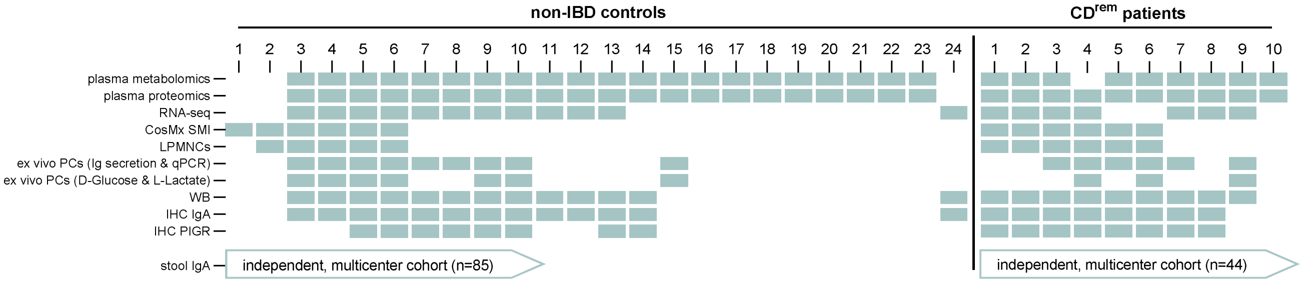

Supplementary Fig. 6 | Patient-Method matrix. Matrix displaying which of the 24 non-IBD control patients (left) and 10 CD^{rem} patients (right) were included in each method in this study.

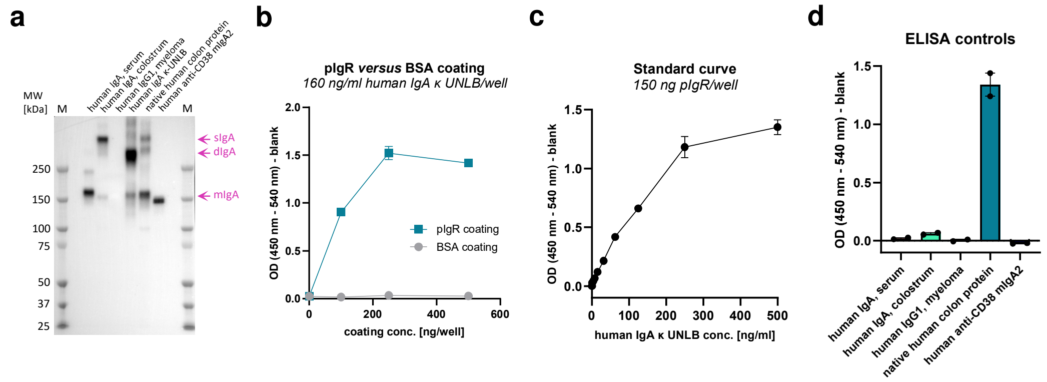

Supplementary Fig. 7 | Establishment of dimeric IgA-specific ELISA. (a) Profiling of different purified human IgA samples and native colon protein by Western blot experiment using the anti-human IgA antibody (Thermo Fisher Scientific #PA1-74395). **(b)** Verification of binding specificity of human IgA κ UNLB (mainly dIgA) to human recombinant pIgR. **(c)** Standard curve of human IgA κ UNLB with coating of 150 ng pIgR/well. **(d)** Validation of binding specificity for dIgA testing native human colon protein (positive control) against various negative control samples (equimolar amounts). **(b)**-**(d)** Each data point represents the mean of technical duplicates with whiskers indicating the range.

Supplementary Table 1. Patient characteristics of plasma and stool samples.

		Non-IBD	CD remission
		**Plasma samples**	
**Metabolomics**	**Total number of patients**	21	9
	**Female/male**	16 (76.2)/5 (23.8)	5 (55.6)/4 (44.4)
	**Age, y**	60.0 (48.0-65.0)	50.0 (38.5-61.0)
	**Colonic involvement**		
	Yes/Unknown	NA	8 (88.9)/1 (11.1)
	**Macroscopic inflammation**		
	Terminal Ileum/ICV/Colon/NA	-/-/-/7 (33.3)	2 (22.2)/1 (11.1)/-/-
	**Medication**		
	5-ASA/ADA/IFX/UPA/UST/ UST+PRED/VDZ/None	-/-/-/-/-/-/-/21 (100.0)	1 (11.1)/1 (11.1)/2 (22.2)/1 (11.1)/2 (22.2)/ 1 (11.1)/1 (11.1)/-
**Proteomics**	**Total number of patients**	21	10
	**Female/male**	16 (76.2)/5 (23.8)	6 (60.0)/4 (40.0)
	**Age, y**	60.0 (48.0-65.0)	47.0 (35.0-60.0)
	**Colonic involvement**		
	Yes/Unknown	NA	9 (90.0)/ 1 (10.0)
	**Macroscopic inflammation**		
	Terminal Ileum/ICV/Colon/NA	-/-/-/7 (33.3)	2 (20.0)/1 (10.0)/-/-
	**Medication**		
	5-ASA/ADA/IFX/UPA/UST/ UST+PRED/VDZ/None	-/-/-/-/-/-/-/21 (100.0)	1 (10.0)/1 (10.0)/2 (20.0)/1 (10.0)/2 (20.0)/ 1 (10.0)/1 (10.0)/1 (10.0)
		**Stool samples**	
**IgA-ELISA**	**Total number of patients**	85	44
	**Female/male**	56 (65.9)/29 (34.1)	30 (68.2)/14 (31.8)
	**Age, y**	35.0 (28.0-56.0)	37.0 (29.0-49.0)
	**Disease manifestation**		
	Colonic/Ileocolonic/ Ileocolonic + uGIT	NA	12 (27.3)/27 (61.4)/5 (11.4)
	**Harvey-Bradshaw Index (HBI)**		
	0/1/2/3/Unknown	NA	10 (22.7)/9 (20.5)/5 (11.4)/3 (6.8)/17 (38.6)
	**CRP, mg/l**		
	< 5/5-17/Unknown	-/-/85 (100.0)	34 (77.3)/1 (2.3)/9 (20.5)
	**Fecal Calprotectin, µg/g**		
	≤ 50/51-99/≥ 100/Unknown	-/-/-/85 (100.0)	25 (56.8)/11 (25.0)/-/8 (18.2)
	**Medication**		
	5-ASA/AZA/AZA+UST/AZA+ UST+BUD+PRED/RZB/TNF/ TNF+5-ASA/TNF+AZA/UST/ VDZ/None	-/-/-/-/-/-/-/-/-/-/ 85 (100.0)	1 (2.3)/2 (4.5)/3 (6.8)/1 (2.3)/2 (4.5)/15 (34.1)/4 (9.1)/7 (15.9)/7 (15.9)/2 (4.5)/-

Values are median (IQR) or n (%). 5-ASA, 5-aminosalicylic acid (mesalazine); ADA, Adalimumab; AZA, Azathioprine; BUD, Budesonide; CD, Crohn's disease; CRP, C-reactive protein; IBD, inflammatory bowel disease; ICV, ileocecal valve; IFX, Infliximab; IQR, interquartile range; NA, not applicable/not available; PRED, Prednisolone; RZB, Risankizumab; TNF, Anti-TNF; uGIT, upper gastrointestinal tract; UPA, Upadacitinib; UST, Ustekinumab; VDZ, Vedolizumab.

Supplementary Table 2. Patient characteristics of native and formalin-fixed colon biopsies.

		Non-IBD	CD remission
		**Colon biopsies**	
**CosMx SMI**	**Total number of patients**	6	6
	**Female/male**	3 (50.0)/3 (50.0)	3 (50.0)/3 (50.0)
	**Age, y**	50.0 (33.5-64.5)	54.5 (35.0-63.0)
	**Colonic involvement**		
	Yes/Unknown	NA	5 (83.3)/ 1 (16.7)
	**Macroscopic inflammation**		
	Terminal Ileum/Colon	-/-	2 (33.3)/-
	**Origin of colon biopsy**		
	C. descendens/C. sigmoideum	3 (50.0)/3 (50.0)	5 (83.3)/1 (16.7)
	**Medication**		
	5-ASA/IFX/UPA/UST/None	-/-/-/-/6 (100.0)	1 (16.7)/2 (33.3)/1 (16.7)/1 (16.7)/1 (16.7)
**IHC IgA**	**Total number of patients**	13	8
	**Female/male**	7 (53.8)/6 (46.2)	3 (50.0)/3 (50.0)
	**Age, y**	54.0 (48.5-67.0)	47.0 (36.8-62.0)
	**Colonic involvement**		
	Yes/Unknown	NA	7 (87.5)/1 (12.5)
	**Macroscopic inflammation**		
	Terminal Ileum/ICV/Colon	-/-/-	2 (25.0)/1 (12.5)/-
	**Origin of colon biopsy**		
	Flexura coli sinistra/	1 (7.7)/	-/
	C. descendens/C. sigmoideum	7 (53.8)/5 (38.5)	6 (75.0)/2 (25.0)
	**Medication**		
	5-ASA/IFX/UPA/UST/	-/-/-/-/-/-/13 (100.0)	1 (12.5)/2 (25.0)/1 (12.5)/1 (12.5)/
	UST+PRED/VDZ/None		1 (12.5)/1 (12.5)/1 (12.5)
**IHC PIGR**	**Total number of patients**	8	8
	**Female/male**	5 (62.5)/3 (37.5)	3 (50.0)/3 (50.0)
	**Age, y**	53.5 (51.5-64.5)	47.0 (36.8-62.0)
	**Colonic involvement**		
	Yes/Unknown	NA	7 (87.5)/1 (12.5)
	**Macroscopic inflammation**		
	Terminal Ileum/ICV/Colon	-/-/-	2 (25.0)/1 (12.5)/-
	**Origin of colon biopsy**		
	C. descendens/C. sigmoideum	4 (50.0)/4 (50.0)	6 (75.0)/2 (25.0)
	**Medication**		
	5-ASA/IFX/UPA/UST/	-/-/-/-/-/-/8 (100.0)	1 (12.5)/2 (25.0)/1 (12.5)/1 (12.5)/
	UST+PRED/VDZ/None		1 (12.5)/1 (12.5)/1 (12.5)
**Transcriptomics**	**Total number of patients**	12	7
	**Female/male**	6 (50.0)/6 (50.0)	4 (57.1)/3 (42.9)
	**Age, y**	58.5 (47.8-68.0)	50.0 (42.0-59.0)
	**Colonic involvement**		
	Yes/Unknown	NA	6 (85.7)/1 (14.3)
	**Macroscopic inflammation**		
	Terminal Ileum/ICV/Colon	-/-/-	1 (14.3)/1 (14.3)/-
	**Origin of colon biopsy**		
	Flexura coli sinistra/	1 (8.3)/7 (58.3)/4 (33.3)	-/6 (85.7)/1 (14.3)
	C. descendens/C. sigmoideum		
	**Medication**		
	5-ASA/IFX/UST/UST+PRED/	-/-/-/-/-/12 (100.0)	1 (14.3)/1 (14.3)/2 (28.6)/1 (14.3)/
	VDZ/None		1 (14.3)/1 (14.3)

**Western Blot**	**Total number of patients**	13	9
	**Female/male**	7 (53.8)/6 (46.2)	5 (55.6)/4 (44.4)
	**Age, y**	54.0 (48.5-67.0)	50.0 (38.5-61.0)
	**Colonic involvement**		
	Yes/Unknown	NA	8 (88.9)/1 (11.1)
	**Macroscopic inflammation**		
	Terminal Ileum/ICV/Colon	-/-/-	2 (22.2)/1 (11.1)/-
	**Origin of colon biopsy**		
	Flexura coli sinistra/ C. descendens/C. sigmoideum	1 (7.7)/7 (53.8)/5 (38.5)	-/7 (77.8)/2 (22.2)
	**Medication**		
	5-ASA/IFX/UPA/UST/ UST+PRED/VDZ/None	-/-/-/-/-/-/13 (100.0)	1 (11.1)/2 (22.2)/1 (11.1)/2 (22.2)/ 1 (11.1)/1 (11.1)/1 (11.1)

Values are median (IQR) or n (%). 5-ASA, 5-aminosalicylic acid (mesalazine); CD, Crohn's disease; IBD, inflammatory bowel disease; ICV, ileocecal valve; IFX, Infliximab; IQR, interquartile range; NA, not applicable/not available; PRED, Prednisolone; UPA, Upadacitinib; UST, Ustekinumab; VDZ, Vedolizumab.

Supplementary Table 3. Patient characteristics of isolated colonic LPMNCs, plasma CRP quantification, and ex vivo differentiated PCs.

		Non-IBD	CD remission
	**Colon biopsies + plasma samples**		
colonic LPMNCs & plasma CRP	**Total number of patients**	5	6
	**Female/male**	3 (60.0)/2 (40.0)	3 (50.0)/3 (50.0)
	**Age, y**	53.0 (36.5-66.0)	54.5 (35.0-63.0)
	**Colonic involvement**		
	Yes/Unknown	NA	5 (83.3)/1 (16.7)
	**Macroscopic inflammation**		
	Terminal Ileum/ICV/Colon	-/-/-	2 (33.0)/-/-
	**Medication**		
	5-ASA/IFX/UPA/UST/None	-/-/-/-/5 (100.0)	1 (16.7)/2 (33.0)/1 (16.7)/1 (16.7)/1 (16.7)
ex vivo PCs (Ig secretion & qPCR)	**Total number of patients**	9	6
	**Female/male**	5 (55.6)/4 (44.4)	5 (83.3)/1 (16.7)
	**Age, y**	63.0 (49.5-67.0)	46.0 (35.0-59.3)
	**Colonic involvement**		
	Yes/Unknown	NA	6 (100.0)/-
	**Macroscopic inflammation**		
	Terminal Ileum/ICV/Colon	-/-/-	1 (16.7)/1 (16.7)/-
	**Medication**		
	5-ASA/IFX/UPA/UST/VDZ/ None	-/-/-/-/-/9 (100.0)	1 (16.7)/1 (16.7)/1 (16.7)/1 (16.7)/1 (16.7)/ 1 (16.7)
ex vivo PCs (D-Glucose/L-Lactate)	**Total number of patients**	7	3
	**Female/male**	5 (71.4)/2 (28.6)	3 (100.0)/-
	**Age, y**	63.0 (53.0-69.0)	35.0 (35.0-58.0)
	**Colonic involvement**		
	Yes/Unknown	NA	3 (100.0)/-
	**Macroscopic inflammation**		
	Terminal Ileum/ICV/Colon	-/-/-	1 (33.3)/-/-
	**Medication**		
	UPA/UST/None	-/-/-	1 (33.3)/1 (33.3)/1 (33.3)

Values are median (IQR) or n (%). 5-ASA, 5-aminosalicylic acid (mesalazine); CD, Crohn's disease; CRP, C-reactive protein; IBD, inflammatory bowel disease; ICV, ileocecal valve; IFX, Infliximab; IQR, interquartile range; LPMNC, lamina propria mononuclear cell; NA, not applicable/not available; PC, plasma cell; UPA, Upadacitinib; UST, Ustekinumab; VDZ, Vedolizumab.

Supplementary Table 4. Active CD patient characteristics of isolated colonic LPMNCs and plasma CRP quantification.

		CD active
	**Colon biopsies**	
**colonic LPMNCs**	**Total number of patients**	8
	**Female/male**	1 (12.5)/7 (87.5)
	**Age, y**	33.0 (25.5-46.3)
	**Colonic involvement**	
	Yes/Unknown	8 (100.0)/-
	**Macroscopic inflammation**	
	Terminal Ileum/Colon	4 (50.0)/7 (87.5)
	**Medication**	
	5-ASA+UST/6-MP+UST/ADA+PRED+MTX/AZA+CZB/	1 (12.5)/1 (12.5)/1 (12.5)/1 (12.5)/
	IFX/None	1 (12.5)/3 (37.5)
	**Plasma samples**	
**plasma CRP**	**Total number of patients**	10
	**Female/male**	3 (30.0)/7 (70.0)
	**Age, y**	33.0 (24.3-45.3)
	**Colonic involvement**	
	Yes/Unknown	10 (100.0)/-
	**Macroscopic inflammation**	5 (50.0)/8 (80.0)
	Terminal Ileum/Colon	
	**Medication**	1 (10.0)/1 (10.0)/1 (10.0)/1 (10.0)/
	5-ASA+UST/6-MP+UST/ADA+PRED+MTX/BUD+CORT+VDZ/	1 (10.0)/2 (20.0)/3 (30.0)
	IFX/UST/None	

Values are median (IQR) or n (%). 5-ASA, 5-aminosalicylic acid (mesalazine); 6-MP, 6-Mercaptopurine; ADA, Adalimumab; AZA, Azathioprine; BUD, Budesonide; CD, Crohn's disease; CORT, Cortisone; CRP, C-reactive protein; CZB, Certolizumab; IFX, Infliximab; IQR, interquartile range; LPMNC, lamina propria mononuclear cell; MTX, Methotrexate; PRED, Prednisolone; UST, Ustekinumab; VDZ, Vedolizumab.

Supplementary Table 5. Composition of the glucose-free high-protein diet (GFHPD) and the isoenergetic control diet (chow) as provided by the manufacturer.

**Diet formulation [%]**	**Isocaloric control diet (chow)**	**Glucose-free high-protein diet (GFHPD)**
Casein	20	60
Brewer's yeast (*Saccharomyces cerevisiae*)	-	2
Corn starch	28	-
Maltodextrin	14.5	-
Sucrose	10	-
Cellulose powder	15	19.5
L-cystine	0.3	0.3
Vitamin premixture	1	1
Minerals and trace elements	6	6
Choline chloride	0.2	0.2
Soybean oil	5	11
**Nutrient profile [%]**	**Isocaloric control diet (chow)**	**Glucose-free high-protein diet (GFHPD)**
Crude protein	17.7	53.4
Crude fat	5.1	11.4
Crude fiber	15.8	20.3
Crude ash	5.4	5.9
Starch	26.9	0.1
Sugar	9.9	-
Nitrogen-free extract (NfE)	51.7	1.9
**Macronutrient composition [kcal%]**	**Isocaloric control diet (chow)**	**Glucose-free high-protein diet (GFHPD)**
Protein	22	66
Fat	14	32
Carbohydrates	64	2
**Physiological fuel value**	13.6 MJ/kg	13.6 MJ/kg

Supplementary Table 6. Sequences of the primer pairs used for RT-qPCR (produced by Metabion international AG (Planegg, Germany)).

Target gene	Forward primer (5'-3')	Reverse primer (5'-3')
**human**		
ACTB	ACATCCGCAAAGACCTGTACG	TTGCTGATCCACATCTGCTGG
CD14	CATCCAGAATCTAGCGCTGC	CAGCGAACGACAGATTGAGG
CD19	TCTTCTGCCTGTGTTCCCTT	GCTGCTCGGGTTTCCATAAG
CD2	GAATGCCTTGGAAACCTGGG	TTCGGGGTCAGTTCCATTCA
CD27	CTGCTCAGTGTGATCCTTGC	GTCAGCGAAGGGTTTGGAAG
CD36	AAAACGGCTGCAGGTCAAC	TCACCACACCAACACTGAGT
CD38	GGAGAAAGGACTGCAGCAAC	CATGTATCACCCAGGCCTCT
CD3E	CGGTGGCCACAATTGTCATA	TCAGGCCAGAATACAGGTCC
CPT1A	GAGAGACAGCAAGCACATCG	ACATCGGCCGTGTAGTAGAG
CR2	GTCCAGTGCTCACATGTCCA	ACTCTGTCTCACATGCTGGC
CS	CAGCTGCAGAAGGAAGTTGG	AGTACACCCAATGCTCGTGA
FCGR3A	GTGTTCCTGGAGCCTCAATG	TGAGGGTGGAGAGGTTTGTC
GYPA	TGGCTGGTGTTATTGGAACG	TCATTGATCACTTGTCTCTGGAT
IGHA1	AAGACCTTCACTTGCACTGC	TTCTTCCAGTCCTCGGCTG
IGHA2	AGGATGTGACTGTGCCCTG	TAGTGGGGTCTTCAACTCGG
IGHD	CGTCAAGCTTTCCCTGAACC	CGCTGTTCTCATCCTTGCTC
IGHG1	AGGACTCTACTCCCTCAGCA	GGTGGGCATGTGTGAGTTTT
IGHG2	GACAAGACAGTTGAGCGCAA	TGTTGGAGACCTTGCACTTG
IGHG3	AGGACTCTACTCCCTCAGCA	CAGGGGTCCGGGAAATCATA
IGHG4	AGGACTCTACTCCCTCAGCA	GGTCCGGGAGATCATGAGAG
IGHM	CAGAATGCGTCCTCCATGTG	GTGGCAGCAAGTAGACATCG
ITGAX	CCAGGAAACAAAGAACCGGAG	GAGGCCGTGAAGTATCTCTGA
JCHAIN	ACAAATGTAAGTGTGCCCGG	CCAGCTCCACTTCTGTAGGA
LDHA	GCACCCAGTTTCCACCATGA	GCACTCTTCTTCAAACGGGC
MS4A1	ACCCATCTGTGTGACTGTGT	AGAAATGGCAGCAAAGAGGC
MT-ATP6	GCCACCCTAGCAATATCAACC	TGTGTTGTCGTGCAGGTAGA
MT-ATP8	CACAAACTACCACCTACCTCC	GGGGCAATGAATGAAGCGAA
MT-CO1	CGTTATCGTCACAGCCCATG	GTTCAACCTGTTCCTGCTCC
MT-CO3	TTTACCACTCCAGCCTAGCC	CTCTGAGGCTTGTAGGAGGG
MT-ND3	GACTACCACAACTCAACGGC	GGGCTCATGGTAGGGGTAAA
MZB1	CGAGTTGGTCTACACGGATG	TGACACCTTCTCTGAGCAGG
NCAM1	AATGTGCCACCTACCATCCAG	AGATGTACTCAGCCTCGTCG
PDK1	GTCACCAGCCAGAATGTTCA	CTCTGTTGGCATGGTGTTCC
PRDM1	GCAGAACGGCAAGATCAAGT	CCGTGTGTACCAGGTAGTGT
PTPRC	ACATCATCACCTAGCAGTTCATG	GGGAAGGTGTTGGGCTTTG
SDC1	GCCGCAAATTGTGGCTACT	GCTGCGTGTCCTTCCAAGT
SLC1A5	CTGTGGGCATCATGTTCCTG	GATGAAACGGCTGATGTGCT
SLC2A1	TGGCATCAACGCTGTCTTCT	CTAGCGCGATGGTCATGAGT
SLC2A3	GGCTGCTTTATGGGACTGTG	CAAAATGGAAGGGCTGCACT
SLC3A2	AGCTACGGGGATGAGATTGG	CCCAGTGGCGGATATAGGAG
SLC7A5	TACTTCACCACCCTGTCCAC	TAGAGCAGCGTCATCACACA
SLC38A1	TTTCAGCCTGGTACGTGGAT	ACAGCAACAATGACAGCCAG
SLC38A2	AGACTGTCTATGCTGTGCCA	AGCCAGACGGACAATGAGAA
SLC38A5	TGCTGCCCATCTATACGGAG	GGGTACAATGCGGAGGTAGA
SPN	CCGGAACCCAGATGAGAACT	AGACTTCAGCTCCTCCATCG
TNFRSF13B	TTTGCAACCATCAGAGCCAG	TTCAACTTCTCCACTCCGCT
TNFRSF17	CCTCCTCTAACATGTCAGCG	TTCATCACCAGTCCTGCTCT
UBC	CCGGGATTTGGGTCGCAG	TCACGAAGATCTGCATTGTCAAG
XBP1_spliced	CTGAGTCCGCAGCAGGTG	AAGGGAGGCTGGTAAGGAAC
XBP1_unspliced	TCAGACTACGTGCACCTCTG	AAGGGAGGCTGGTAAGGAAC
**murine**		
Cd19	TCTGGCTGTTGAGAACTGGT	GGAGAGCACATTCCCGTACT
C1qbp	CGCTCTGCACACGGAAGGAG	CGGCCTCATCTTCGTGTCCA
Igha	ACCGTAAACTTCCCACCTGC	GGACGGCGTTAGAGTCATGT
mt-Nd3	AAGCAAATCCATATGAATGCG	TTTGAATTGCTCATGGTAGTGG
Prdm1	GTCGCGGAGACGCAAG	CCACGCCAATAACCTCTTTG

Ptprc	TCATGGTCACACGATGTGAAGA	AGCCCGAGTGCCTTCCT
Sdc1	AGGATGGAACTGCCAATCAG	ATCCGGTACAGCATGAAAGC
Tnfrsf17	GGTGCTCTGGATCTTCTTGG	CCGTAGTCACCCGTTTTTGT

Supplementary Table 7. Details of applied primary and secondary antibodies.

Primary antibody	Species	Company	Working concentration
Anti-α-Tubulin (clone 11H10) (#2125)	rabbit	Cell Signaling Technology, Danvers, MA	8 ng/ml (WB)
Anti-β-Actin (#4967)	rabbit	Cell Signaling Technology, Danvers, MA	0.009 µg/ml (WB)
Anti-human BCMA/TNFRSF17 (clone E6D7B) (#88183)	rabbit	Cell Signaling Technology, Danvers, MA	0.78 µg/ml (WB)
Anti-human IgA (clone AD3) (#LS-B3728)	mouse	LifeSpan Biosciences, Seattle, WA	0.5 µg/ml (IF, IHC)
Anti-human IgA HRP-conjugated (#PA1-74395)	goat	Thermo Fisher Scientific, Waltham, MA	1:8000 (ELISA, WB)†
Anti-human IgG HRP-conjugated (#62-8420)	goat	Thermo Fisher Scientific, Waltham, MA	0.25 µg/ml (WB)
Anti-human IgM (#A80-100A)	goat	Bethyl Laboratories, Montgomery, TX	0.5 µg/ml (WB)
Anti-human PIGR (clone EPR23314-256) (#ab275020)	rabbit	Abcam, Cambridge, United Kingdom	4.82 µg/ml (IHC)
Anti-mouse B220 BV785™ (clone RA3-6B2) (#103245)	rat	BioLegend, San Diego, CA	1 µg/ml (FC)
Anti-mouse CD138 BV711™ (clone 281-2) (#563193)	rat	BD Biosciences, Franklin Lakes, NJ	1 µg/ml (FC)
Anti-mouse CD16/32 (clone 93) (#101301)	rat	BioLegend, San Diego, CA	2 µg/ml (FC)
Anti-mouse IgA (alpha chain) (#PAB9360)	rabbit	Abnova, Taipei, Taiwan	125 ng/ml (WB)
Anti-mouse IgA HRP-conjugated (#A90-103P)	goat	Bethyl Laboratories, Montgomery, TX	4 µg/ml (IHC)
Anti-mouse IgG1 BV421™ (clone RMG1-1) (#406615)	rat	BioLegend, San Diego, CA	0.125 µg/ml (FC)
Anti-P32/GC1qR (clone EPR8871) (#ab131284)	rabbit	Abcam, Cambridge, United Kingdom	2.5 µg/ml (IF)
**Secondary antibody**		**Company**	**Working concentration**
Anti-goat Igs HRP-conjugated (#P0449)		Agilent Technologies, Santa Clara, CA	0.25 µg/ml (WB)
Anti-mouse IgG HRP-conjugated (#7076)		Cell Signaling Technology, Danvers, MA	0.153 µg/ml (WB)
Anti-rabbit IgG HRP-conjugated (#7074)		Cell Signaling Technology, Danvers, MA	19.25 ng/ml (WB)
EnVision+ anti-mouse HRP (#K400111-2)		Agilent Technologies, Santa Clara, CA	Not applicable
EnVision+ anti-rabbit HRP (#K400311-2)		Agilent Technologies, Santa Clara, CA	Not applicable
Goat anti-mouse IgG Alexa Fluor® 488 (#4408)		Cell Signaling Technology, Danvers, MA	4 µg/ml (IF)
Goat anti-rabbit IgG Alexa Fluor™ 546 (#A-11035)		Thermo Fisher Scientific, Waltham, MA	8 µg/ml (IF)

ELISA, enzyme-linked immunosorbent assay; FC, flow cytometry; IF, immunofluorescence; IHC, immunohistochemistry; WB, western blot. † If stock concentration was not provided by the manufacturer, dilution from stock solution is indicated.

Supplementary References

1. Elmentaite, R. *et al.* Cells of the human intestinal tract mapped across space and time. *Nature* **597**, 250–255 (2021).
